# Pseudotumour cerebri associated with mycoplasma pneumoniae infection and treatment with levofloxacin: a case report

**DOI:** 10.1186/s12887-018-1371-9

**Published:** 2019-01-05

**Authors:** Laura Maffeis, Robertino Dilena, Sophie Guez, Francesca Menni, Cristina Bana, Silvia Osnaghi, Giorgio Carrabba, Paola Marchisio

**Affiliations:** 1 Pediatric Highly Intensive Care Unit, Fondazione IRCSS Ca’ Granda Ospedale Maggiore Policlinico, Milan, Italy; 2 Service of Pediatric Neurophysiology , Unit of Clinical Neurophysiology, Fondazione IRCCS Ca’ Granda, Ospedale Maggiore Policlinico, Milan, Italy; 3 Department of Ophthalmology, Fondazione IRCCS Ca’ Granda Ospedale Maggiore Policlinico, Milan, Italy; 4 Division of Neurosurgery, Fondazione IRCCS Ca’ Granda Ospedale Maggiore Policlinico, Milan, Italy; 5 Pediatric Highly Intensive Care Unit, Fondazione IRCSS Ca’ Granda Ospedale Maggiore Policlinico, and a Department of Pathophysiology and Transplantation, Università degli Studi di Milano, Milan, Italy

**Keywords:** Intracranial hypertension, Pseudotumour cerebri syndrome, Mycoplasma pneumoniae, Levofloxacin, Paediatric

## Abstract

**Background:**

Idiopathic intracranial hypertension (IIH), also known as pseudotumour cerebri syndrome (PTCS), is characterized by the presence of signs and symptoms of raised intracranial pressure without evidence of any intracranial structural cause and with normal cerebrospinal fluid microscopy and biochemistry.

Obesity, various systemic diseases and endocrine conditions, and a number of medications are known to be risk factors for PTCS. The medications commonly associated with PTCS are amiodarone, antibiotics, corticosteroids, cyclosporine, growth hormone, oral contraceptives, vitamin A analogues, lithium, phenytoin, NSAIDs, leuprolide acetate, and some neuroleptic drugs. In relation to antibiotics, quinolones may cause intracranial hypertension, and most reported cases of quinolone-induced intracranial hypertension were associated with nalidixic acid, ciprofloxacin, ofloxacin, or pefloxacin. Literature reports of levofloxacin-induced PTCS are rare. Some authors recently hypothesized that Mycoplasma pneumoniae may trigger PTCS.

**Case presentation:**

We report on a 14-year-old overweight White Italian boy who suffered headache, diplopia, and severe bilateral papilloedema after a Mycoplasma pneumoniae infection, exacerbated on levofloxacin intake. A spontaneous improvement in headache and a reduction in diplopia was seen during hospitalisation. Oral acetazolamide therapy led to the regression of papilloedema in about five months. No permanent eye damage has been observed in our patient to date.

**Conclusions:**

PTCS pathophysiology may be multifactorial and its specific features and severity may be a consequence of both constitutional and acquired factors interacting synergistically. It may be useful for paediatricians to know that some antibiotics may have the potential to precipitate PTCS in patients who already have an increased CSF pressure due to a transitory imbalanced CSF circulation caused by infections such as Mycoplasma pneumoniae, with headache being the first and most sensitive, but also the least specific, symptom.

**Electronic supplementary material:**

The online version of this article (10.1186/s12887-018-1371-9) contains supplementary material, which is available to authorized users.

## Background

Idiopathic intracranial hypertension (IIH), also known as pseudotumour cerebri syndrome (PTCS), is defined as raised intracranial pressure in the absence of underlying causes such as intracranial mass lesions, cerebral malformations, CNS infections, cerebral venous sinus thrombosis, or hydrocephalus [1, 2].

The incidence of PTCS in children has been estimated as 0.5–0.9 per 100,000 children per year [1, 3], although this estimate is based on small or retrospective studies. Recently, Matthews et al. published a national prospective population-based cohort study that is a prospective survey of all cases of paediatric PTCS in the United Kingdom and establishes, for the first time, reliable estimates of age-specific, sex-specific and weight-specific annual incidence rates [4].

The classic symptoms of PTCS are headache, nausea, tinnitus, blurring of vision and diplopia. In 1937 Dandy defined the diagnostic criteria for PTCS [5], and in 2013 Friedman et al. published revised criteria which categorise PTCS as “definitive”, “probable”, or “suggestive of PTCS” [6].

The severity of papilloedema may be variable and the eyes may be asymmetrically involved. Currently, there is no diagnostic-therapeutic consensus algorithm, since there are no randomised studies that allow evidence-based treatment. The management of PTCS remains controversial. The current trend is close clinical monitoring of signs and symptoms. The therapeutic approach is based on clinical severity, with particular attention to the degree of eye involvement [1–3, 7].

The prognosis of PTCS is generally good. With early diagnosis and treatment, most children have complete resolution of symptoms. Nevertheless, complications of persistent papilloedema may lead to loss of visual acuity or even blindness. Ophthalmological assessment and monitoring is therefore strongly recommended by all authors.

The exact pathogenesis of PTCS is unknown and many associated aetiologies are reported in literature. Mosquera Gorostidi et al. analysed a total of 12 children with PTCS and described a possible association with Mycoplasma pneumoniae [3]. These authors hypothesized that M. pneumoniae may trigger intracranial hypertension and cause recurrences at later stages.

An iatrogenic hypothesis for PTCS has also been proposed in the literature and several medications have been associated with PTCS. The oldest known association is with vitamin A and retinoids. Other described associations are with lithium, steroids, reproductive hormones (progestins, oestrogens, testosterone, contraception or hormone supplementation therapy), thyroid replacement therapy, recombinant human growth hormone, non-steroidal anti-inflammatory drugs and Nonan and Neem extract, used as a supplement for infants in southern India [8]. Antibiotics (tetracyclines (tetracycline, minocycline and doxycycline), sulfamethoxazole, gentamicin, cephalexin and quinolones) are also reported as a possible cause of PTCS. Most reported cases of quinolone-induced intracranial hypertension were associated with nalidixic acid [9–12], ciprofloxacin [13], ofloxacin [14], or pefloxacin [15]. Literature reports of levofloxacin-induced PTCS intracranial hypertension are rare [16, 17].

## Case presentation

A 14-year-old White Italian boy came to our Emergency Unit with a headache that had worsened over 20 days together with blurred vision and diplopia over the previous 10 days. His past history was negative for significant morbidities. He reported a recent episode of fever associated with cough, which coincided with the onset of headache. For this respiratory infection he had started taking levofloxacin 500 mg once a day one week before coming to our attention but had stopped taking it after three days due to worsening headache. This headache was initially associated with daytime somnolence, myalgia and arthralgia. The somnolence and arthralgia underwent rapid and spontaneous regression, with subsequent appearance of blurred vision.

**Fig. 1:**
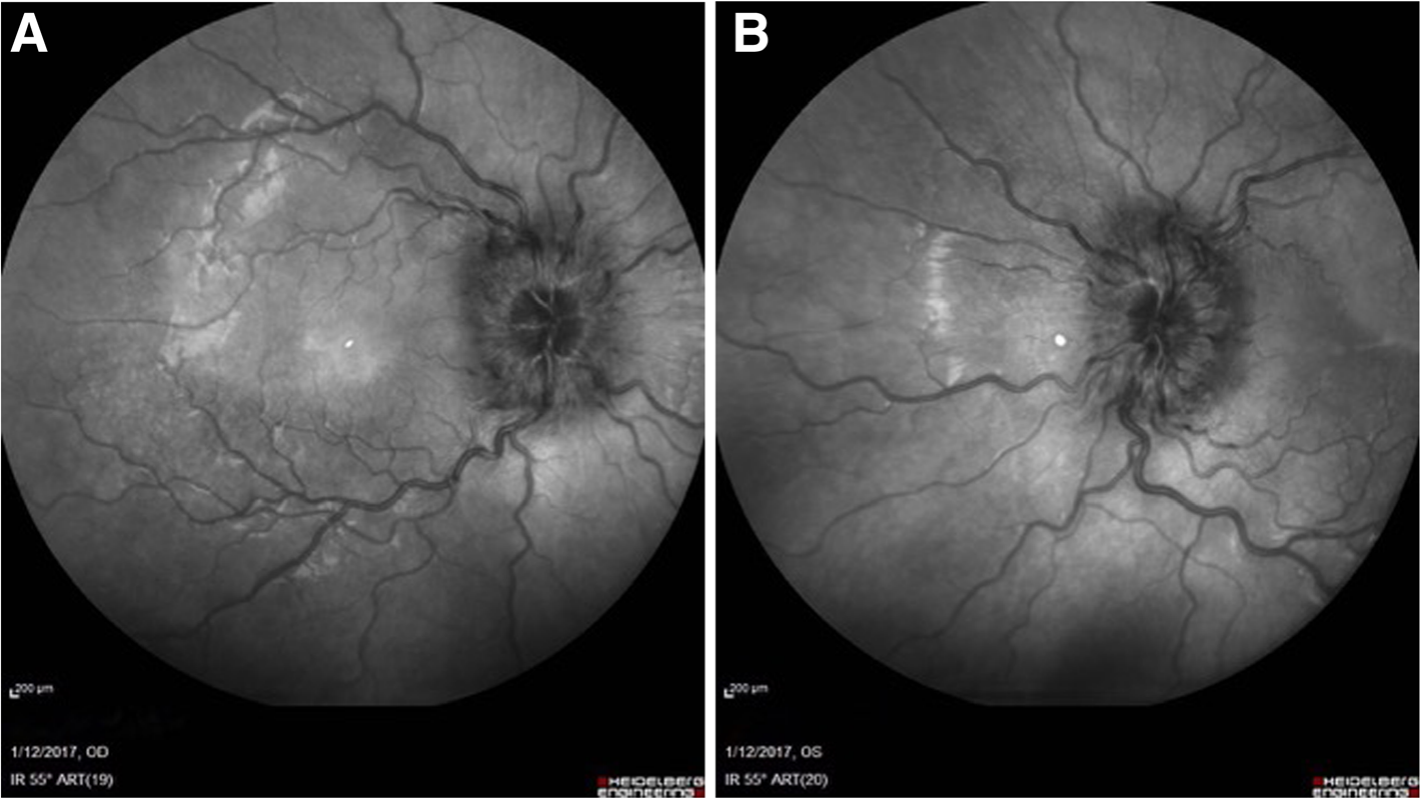
**a**-**b** IR fundus photography. Elevated disc, blurred margins and vessel tortuosity was found at the first ophthalmological visit in both eyes (A-right eye, B-left eye)

The physical examination revealed an alert adolescent with weight of 66 kg (75th -90th percentile) [18], height of 169 cm (50th–75th percentile) [19] and body mass (BMI) of 23.1 kg/m2 (85th–95th percentile) [19]. The general examination was normal. The neurological examination was normal except for a right eye abduction deficit. Eye examination showed a normal visual acuity (10/10) in both eyes with normal colour vision and pupillary light responses, but a fundus examination revealed severe bilateral papilloedema with elevated disc, hyperaemia, blurred margins and vessel tortuosity in both eyes (Fig. 1a-b). Lancaster red-green test confirmed a right abducens nerve palsy, and campimetry showed a restricted visual field with external right muscle deficiency on the right side. Cranial neuroimaging (CT and MRI) showed a normal brain parenchyma with no evidence of hydrocephalus, mass, structural lesion, or abnormal meningeal enhancement. MRI neuroimaging showed oedema of both optic nerves with a tapered appearance of the right optic nerve. Venography was not performed, but an angio-MRI of the cerebral circulation was normal. Visual evoked cortical potentials were normal. A 24-h Ambulatory Blood Pressure Monitoring was negative.

Blood tests showed high M. pneumoniae IgM (15.00 AU/ml, normal range 0–9) and normal M. pneumoniae IgG levels (3.89 AU/ml, normal range 0–9) suggesting a recent infection, with normal white blood cell indices and negative C-reactive protein. Clarithromycin was then prescribed for 14 days without any adverse effects.

Serological screening for Coxsackie, Parvovirus, ECHO virus, Adenovirus, Cytomegalovirus (CMV), Epstein-Barr Virus (EBV), Herpes Simplex Virus 1 (HSV1), and Herpes Simplex Virus 2 (HSV2) excluded recent infections. Thyroid function was normal. Antinuclear antibodies (ANA), anti-double stranded DNA (dsDNA), ENA screening and rheumatoid factor were negative.

During hospitalisation we observed a complete and spontaneous regression of headache and an initial spontaneous reduction in diplopia within a few days. Oral prednisone 50 mg/day (0.75 mg/kg/day) was administered for a week and ocular fundus was monitored.

Since severe bilateral papilloedema persisted one week after the first assessment, lumbar puncture was performed with the patient sedated and relaxed in lateral recumbent position. Opening cerebrospinal fluid (CSF) pressure measured with a standard manometer was 20 cm H_2_O and closing pressure was 19 cm H_2_O. These CSF pressure values have traditionally been considered borderline, but are within normal range according to a recent study in children [20].

CSF biochemical tests and cultures were negative. HSV1, HSV2, VZV, HHV6, CMV, Neisseria, Haemophilus, Streptococcus pneumoniae, Streptococcus B group, *Escherichia coli*, Listeria and *Cryptococcus neoformans*, Parvovirus, Adenovirus, EBV DNA and Enterovirus and Parechovirus RNA PCR were negative. CSF oligoclonal bands were absent on CSF and blood tests.

**Fig. 2:**
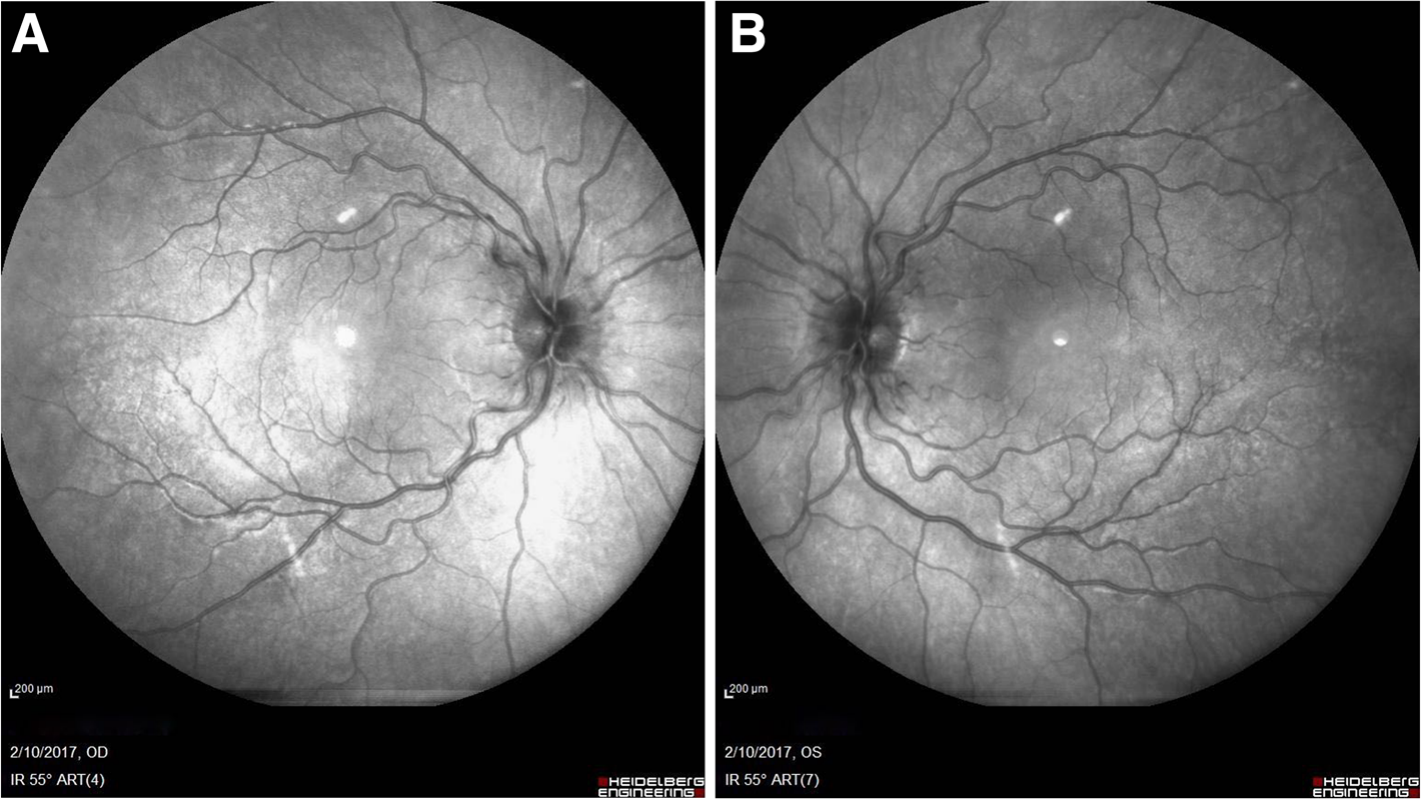
**a**-**b** IR fundus photography. Reduction in the papilloedema was found after one month of acetazolamide: the margins of the disc appear sharper but the vessel tortuosity persists (A-right eye, B-left eye)

**Fig. 3:**
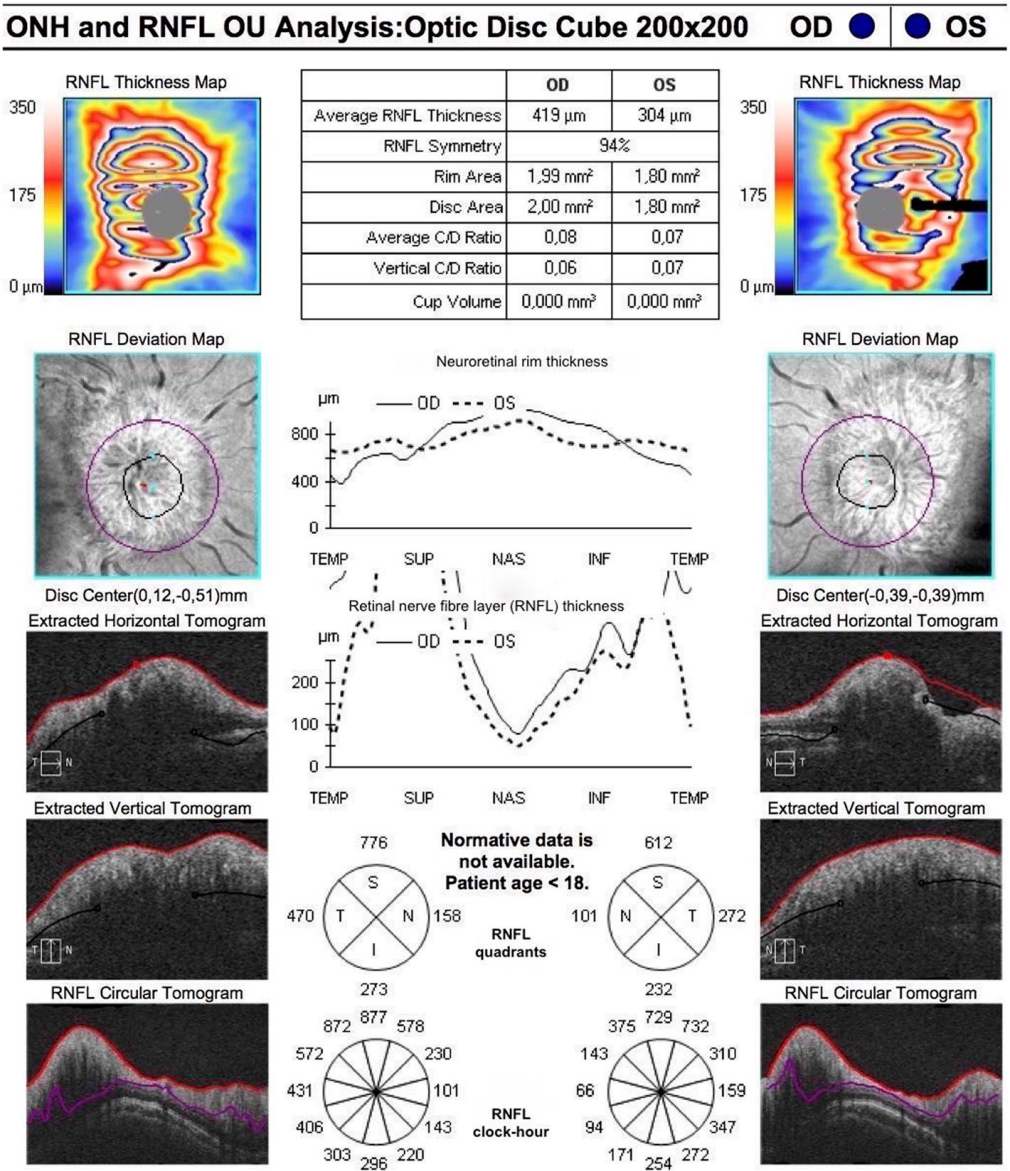
OCT performed at the first visit. An abnormal increase in RNFL thickness was observed

**Fig. 4:**
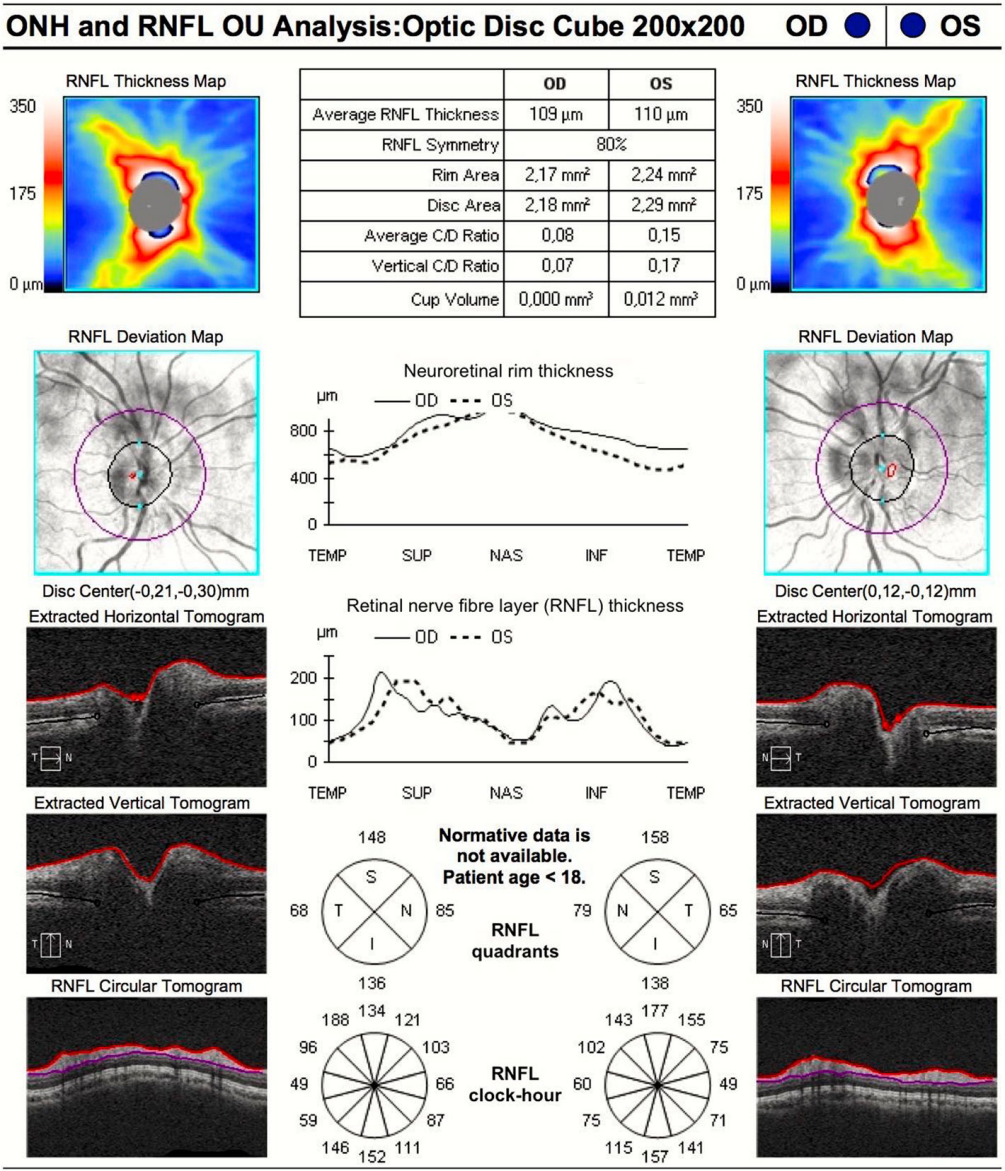
OCT performed after three months of acetazolamide. A dramatic reduction in average RNFL thickness was documented

Oral acetazolamide (1 g divided twice daily) was introduced to accelerate recovery. A gradual further improvement in diplopia was seen during hospitalisation (Fig. 2a-b). Ophthalmological, neurological and neurosurgical follow up was continued after discharge. The patient gradually improved, with complete resolution of the right abducens nerve palsy in one month and resolution of papilloedema in three months (Figs. 3 and 4). For this reason, acetazolamide was gradually reduced and stopped on resolution of the papilloedema (see Additional file 1).

## Discussion and conclusions

This case involved a Mycoplasma pneumoniae infection, probably occurring before the onset of headache and disturbed vision. Headache, diplopia and blurred vision were preceded by respiratory symptoms (cough) and systemic symptoms (such as fever, myalgia and arthralgia), which may be related to M. pneumoniae infection. As previously described [3], our case suggests that M. pneumoniae may trigger PTCS. Furthermore, in our case the already present headache dramatically worsened after administration of levofloxacin. In fact, the patient decided to stop taking levofloxacin after just three days.

Quinolone-induced intracranial hypertension is well described in the literature. The onset of pseudotumour cerebri with quinolones is variable, and can occur after a few days or several weeks of treatment. In this case, the clinical course suggests that M. pneumoniae infection and levofloxacin therapy have a synergic role in precipitating the most severe symptoms of raised intracranial pressure.

The delay of lumbar puncture was due to the fact that during the first days of hospitalization the symptoms (headache and diplopia) were dramatically and spontaneously reduced. For the same reason, a short therapy with oral prednisone was attempted with the aim of promoting the reduction of the symptoms which had already spontaneously started.

A 24-h Ambulatory Blood Pressure Monitoring was performed because a positive family history for essential hypertension at a young age was reported.

CSF oligoclonal bands were absent on CSF and blood tests. It supported the absence of a neurological inflammatory disease.

Although lumbar puncture was performed later, when the symptoms had already improved, we hypothesize that the CSF pressure must have been higher when the symptoms peaked and the papilloedema probably developed.

A CSF opening pressure (OP) of ≥28 cm H_2_O [21] is considered a diagnostic criterion for PTCS in children. However, it has been proposed that a diagnosis of “probable” PTCS can be made with an OP < 28 cm H_2_O, if the other diagnostic criteria are met [22]; OP values must always be interpreted within the clinical context as a whole. In our patient OP was 20 cm H_2_O, but the presence of 1) clinical signs and symptoms of raised intracranial pressure (headache, diplopia, papilloedema and abducent nerve palsy) without additional abnormal neurological signs 2) normal magnetic resonance imaging and 3) unremarkable examination of CSF constituents are supportive for a diagnosis of “probable” PTCS, according to the definition of probable PTCS given by Tibussek et al. [22].

Treatment principles for “probable” PTCS should be similar to those used for demonstrated PTCS [22].

Finally, our case suggests that PTCS pathophysiology may be multifactorial and its specific features and severity may be a consequence of different factors interacting synergistically. This observation needs to be verified in larger studies, but it may be useful for paediatricians to know that some antibiotics may have the potential to precipitate PTCS in patients who already have an increased CSF pressure due to a transient imbalanced CSF circulation caused by infections such as M. pneumoniae, with headache being the first and most sensitive, but also least specific, symptom.

This case also confirmed the importance of a multidisciplinary team including paediatricians, paediatric neurologists, ophthalmologists and neurosurgeons to ensure the good management of PTCS and its complications. Although the prognosis is good in most cases, serial ophthalmological evaluation is required in order to monitor the evolution of papilloedema and preserve visual function.

## Additional file

Additional file 1: Timeline Table. (DOCX 15 kb)

## Abbreviations

CNS: Central nervous system
CSF: Cerebrospinal fluid
CT: Computerized tomography
IIH: Idiopathic intracranial hypertension
MRI: Magnetic resonance imaging
NSAIDs: Nonsteroidal anti-inflammatory drugs
PTCS: Pseudotumour cerebri syndrome
