# Supplementary material for: Pseudotumour cerebri associated with mycoplasma pneumoniae infection and treatment with levofloxacin: a case report
Source: BMC Pediatr. 2019 Jan 5;19:4. doi: 10.1186/s12887-018-1371-9 (PMC6320575; doi:10.1186/s12887-018-1371-9)
Supplement: Supplementary file 1 — Timeline Table. (DOCX 15 kb) [file 12887_2018_1371_MOESM1_ESM.docx]

**Timeline Table**

| **Before 12/2016** | Personal past history was negative for significant morbidities. | | | | |  |  |
| --- | --- | --- | --- | --- | --- | --- | --- |
| **Dates** |  | **Testing** | | | **Interventions** |  |  |
| **12/20/2016** | Episode of fever and cough with headache, daytime somnolence, myalgia and arthralgia | None | | | Paracetamol and NSAIDS |  |  |
| **12/31/2016** | Persistence of fever and cough | None | | | Levofloxacin 500 mg once daily |  |  |
| **01/03/2017** | Worsening headache | None | | | Stop levofloxacin |  |  |
| **01/05/2017** | Diplopia, blurred vision and persistence of headache | Ophthalmological visit+ fundus examination (11/01) 🡪 papilloedema | | | Access to Emergency Unit: urgent blood examination(11/01), cerebral TC (11/01) and neurological assessment 🡪 admission to Pediatric Highly Care Unit |  |  |
| **01/12/2017** | Diplopia, papilloedema | - Serological screening for viral and bacterial infections, thyroid function and autoantibodies screening (12/01) - MRI neuroimaging and angio-MRI (12/01) - Ophthalmological assessment (12/01, 16/01): eye examination. OCT, fundus examination, Lancaster test, campimetry - 24-hour Ambulatory Blood Pressure Monitoring(17/01) - Neurological examination (13/01, 17/01) and visual evoked cortical potential (17/01) - Neurosurgical evaluation (18/01) | | | Clarithromycin 500 mg twice daily for 14 days  Oral prednisone (0,75 mg/kg/day) for a week |  |  |
| **01/23/2017** | Persistence of papilloedema, resolution of headache and initial spontaneous reduction in diplopia | | - Ophthalmological visit (23/01) - Neurosurgical second evaluation (23/01) - Lumbar puncture (26/01) | Oral acetazolamide 1 g twice daily | |  |  |
| **02/10/2017** | Ophthalmological follow up visit n.1  Neurological follow up visit n.1 | | Eye examination, Fundus examination and OCT, campimetry, Lancaster test | Reduction of acetazolamide (250 mg twice daily) | |  |  |
| **04/05/2017** | Ophthalmological follow up visit n.2  Neurological follow up visit n.2 | | Eye examination, Fundus examination and OCT, campimetry and Lancaster test | Stop acetazolamide | |  |  |
| **10/12/2017** | Ophthalmological follow up visit n.3 | | Eye examination, Fundus examination and OCT | No interventions | |  |  |
| **10/19/2017** | Neurological follow up visit n.3 | |  | No interventions | |  |  |
| **04/12/2018** | Ophthalmological follow up visit n.4 | | Eye examination, Fundus examination and OCT | No interventions | |  |  |
| **04/12/2018** | Neurological follow up visit n.4 | |  | No interventions | |  |  |
